# Diagnostic knowing in general practice: interpretative action and reflexivity

**DOI:** 10.1080/02813432.2019.1663592

**Published:** 2019-09-11

**Authors:** Kirsti Malterud, Susanne Reventlow, Ann Dorrit Guassora

**Affiliations:** a The Research Unit for General Practice and Section of General Practice, Department of Public Health, University of Copenhagen, Copenhagen, Denmark;; b Research Unit for General Practice, NORCE Norwegian Research Centre, Bergen, Norway;; c Department of Global Public Health and Primary Care, University of Bergen, Norway

**Keywords:** Diagnosis, knowledge, hermeneutics, decision making, problem solving, general practice

## Abstract

**Background:** Getting the right diagnosis is supposed to provide an explanation of a patient’s health problem and inform health care decisions. As a core element of clinical reasoning, diagnosis deserves systematic and transparent analysis. Conceptual tools can make doctors become aware of and explore diagnostic knowing.

**Methods:** We demonstrate diagnostic knowing analysed as interpretative and contextualised activity. Our analysis is based on Lonergan’s theory of knowing, constituting the cognitive structures as experiencing, understanding, and judging, in a general practice case.

**Findings:** Analysis makes the complexity of diagnostic knowing in this context more transparent, in this case concluding with four diagnostic labels: a corn, constipation, headache and atrial fibrillation. We demonstrate how a medically significant diagnosis does not necessarily evolve deductively from complaints. The opening lines from the patient give ideas of where to look for possible explanations – questions for understanding – rather than diagnostic hypotheses. Such questions emerge from the GP’s experiences from meeting the patient, including imaginations and interpretations. When ideas and questions regarding diagnoses have been developed, they may be judged and subjected to reflection. Questioning may also emerge as transitory concerns, not extensively ruled out. Lonergan’s theory demonstrated a strong fit with these aspects of diagnostic knowing in general practice.

**Implications:** Analysis demonstrated systematic, transparent approaches to diagnostic knowing, relevant for clinical teaching. We argue that an interpretative understanding of diagnosis can change clinical practice, complementing hypothetico-deductive strategies by recognising additional substantial diagnostic modes and giving access to scholarly reflection.

**Key Points**
- Diagnosis is a core element of clinical reasoning, deserving systematic and transparent analysis beyond hypothetico-deductive reasoning or pattern recognition
- Diagnostic knowing in general practice is a special instance of all human knowing with subjectivity, interpretation and reflexivity as essential elements
- Lonergan’s theory for knowing based on experiencing, understanding, and judging allowed us to map, decode and recognise advanced acts of clinical reasoning
- We share our experiences of how these concepts gave us a tool for systematic analysis of the complexities taking place in the GP’s office on an ordinary day

Peter (63), a recently retired teacher, comes to see his regular general practitioner, whom he has consulted for various health problems the last 14 years. According to the appointment book, Peter’s reason for today’s visit is constipation. Systematic analysis of the diagnostic challenges unfolding from this point of departure reveals, however, more complex reasoning than presumed.

## Introduction

Getting the right diagnosis is a key task in health care, supposed to provide an explanation of a patient’s health problem and inform subsequent decisions and treatment [1]. The term diagnosis comes from the Greek word gnosis, meaning knowledge. *Diagnosis* is the act of identifying a disease, illness or problem by examining someone or something, and also a statement or conclusion that describes the reason for a disease, illness or problem [2]. Hence, diagnosis includes the process of establishing diagnostic knowing, as well as its sources and outcomes [3]. Daly calls attention to methodical ways of analysing the practices of clinical reasoning, with knowing (diagnosis, prognosis) and doing (therapeutic decisions) for the individual patient [4]. Here, we shall explore *diagnostic knowing*.

Diagnostic knowing is *context dependent*. McWhinney argued that although general principles of medical problem solving are common, each discipline has its own way of applying them [5]. He demonstrated how specific clinical strategies have been elaborated in primary care, corresponding to the particular morbidity pattern with undifferentiated conditions, illness presenting in early stages, the continuing doctor–patient relationship and time pressure. As clinicians and researchers, the authors have previously explored different aspects of clinical knowledge [6–9]. We understand diagnosis as socially constructed phenomena, surpassing the lists of disease classifications [3]. In this article, we study diagnostic knowing as cognitive and social action in the general practice context.

Elstein et al. presented the *hypothetico-deductive model* as standard clinical reasoning, where the clinician generates a limited number of hypotheses early in the workup guiding the subsequent data collection [10]. In familiar situations, however, experts frequently base their reasoning on *pattern recognition*, comparing and matching the case to a specific instance [11]. We support previous points of view [6,8,9,12–15] suggesting that diagnostic knowing in general practice involves cognitive modes beyond these two classical strategies. Still, the principles of reasoning from complex knowledge sources are poorly understood. As a core element of clinical reasoning within a knowledge-based practice, diagnosis deserves systematic, transparent analysis.

For this purpose, appropriate theoretical frameworks are needed. Leder argues that clinical medicine is not a purified science but, as a hermeneutical enterprise, involves *interpretation* of texts, although well-articulated interpretation models are rarely seen [16]. Engebretsen et al. recommend ‘to make explicit the often implicit interpretational work involved when scientific evidence, clinical expertise and patient preferences are combined’ [17]. Below, we present theoretical perspectives underlying a specific interpretative and reflexive understanding of diagnostic knowing and demonstrate its potentials to understand clinical reasoning.

## Objective and method

Engebretsen et al. introduced the Canadian theologian *Bernard J. F. Lonergan* (1904–1984) [18] in discussions of evidence-based clinical decision making and uncertainty in emergency medicine [17,19]. Lonergan presented his theory of knowing as universal for all domains of insight, dealing with human knowing in general. The contributions from Engebretsen et al. inspired us to situate our analysis of diagnostic knowing in general practice. We have found Lonergan’s theory suitable to support analysis of diagnosis conceived as an interpretative activity in this context.

**Figure 1.**
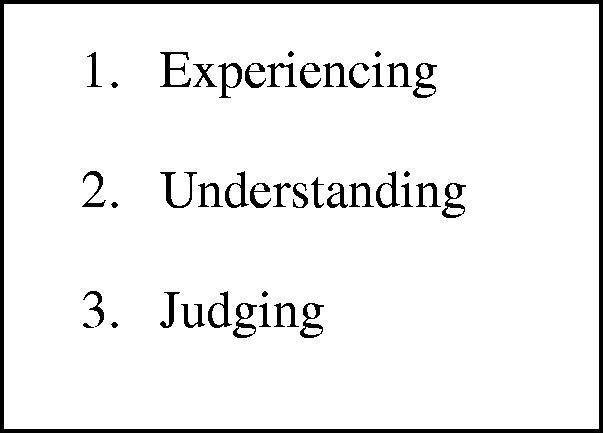
Lonergan’s theory of knowing: a dynamically ordered sequence of three levels of activity, constituting the structure of cognition.

Lonergan understands *insight* as a human act of understanding which requires self-awareness and reflexivity. Analysis of the elements of interpretation allows the general conditions, functions and outcomes of the insight to be thoroughly explored and discussed [18, p.3]. Lonergan’s primary concern is not the known but the knowing as *subjective action* (p.12). Flanagan [20] and Daly [4] summarise Lonergan’s theory of knowing as a dynamically ordered sequence of three levels of activity, constituting the structure of cognition: (1) experiencing, (2) understanding, and (3) judging. Lonergan’s theory also includes a forth element (deliberation), which is not always relevant for diagnostic knowing and therefore not further discussed here (Figure 1).

Engebretsen et al. elaborate these concepts [19], suggesting that *experiencing* is an active process of prereflexive imagination, that *understanding* involves transformation of sensations into ideas, concepts and questions, while *judging* is an argumentative act of weighing the trustworthiness of the understanding. Flanagan emphasise that the three levels are dynamically interrelated by the wondering of the knower [20]. *Inverse insight* is a specific type of knowing [18, pp. 43–50] where the knower realizes that s/he has been asking the wrong question and that the anticipations on which the question was based, were mistaken.

Below, we apply Lonergan’s theory and concepts for analysis of diagnostic knowing on a case from general practice selected for this purpose. Our presentation is a theoretical analysis exploring theory and concepts for systematic and reflexive analysis of diagnostic knowing, not a case study where empirical findings would be highlighted.

The actual case is framed as a consultation in which the patient Peter sees his GP, Kirsti. The case embodies exemplary properties of a general practice consultation (a long-term relationship and a complex presentation of several complaints within a limited time). Another exemplary aspect is the GP’s efforts to prioritise time and energy invested in the different complaints, whilst concurrently attending to the doctor–patient relationship. For reasons of confidentiality, we constructed the case assembling medical and demographic details from several occurrences, arguing that a general practice consultation with comparable elements of diagnostic knowing is familiar and frequently occurring.

## The case

Our case refers to a consultation in Bergen/Norway, where the first author (KM) was a GP for decades. The majority of Norwegians are listed with a GP, tending to use the same GP for many years (average: 7.7 years) [21]. The GP holds considerable previous knowledge about the patient, who may typically present multiple apparently equally prioritised complaints, some not very differentiated. Within the frame of the average consultation time (15 minutes), several conclusions are reached. The patient and the doctor meet again in the not-so-distant future.

Peter (age 63) sits in the waiting room to see Kirsti, his regular GP, whilst Kirsti browses his medical record. Peter is a married, recently retired teacher who has been on Kirsti’s list for the last 14 years. She knows him and his previous medical conditions with prescriptions and follow-ups. From the record, Kirsti briefly reiterates Peter’s hip arthritis (moderate pain), hypertension (stable on medication), psoriasis (periodic outbreaks) and oesophageal hernia (increasing symptoms last year). She also recalls Peter’s usual demeanour—a positively-minded man with a slightly long-winded conversational style, with whom she has always had a good relationship. The appointment list announces that Peter visits for constipation. Kirsti reminds herself about potential follow-up needs related to his chronic conditions. Saying hello to Peter, she gets a feeling that he looks a little more distressed than usual.

Peter presents his constipation with a few words. He says that he goes to the toilet to empty his bowels less often than usual, leading to stomach pain. Before Kirsti gets to expand upon his symptoms, Peter mentions headache and problems with a toe. Kirsti’s awareness is fed by a mixture of organising assignments (where to start and how to proceed) and her perception of Peter (perhaps more concerned than usual). Several ideas in different directions, not very manifestly, appear: Are Peter’s current complaints and appearance associated with his established diseases or medication? Do any of these symptoms and signs indicate serious disease, requiring urgent action? Are all the presented complaints parts of Peter’s agenda today, or do some of them just represent a casual chat? How should she organise the consultation? Which of the complaints require undressing for examination? What could appropriately be postponed to later consultations?

Kirsti reviews Peter’s medication - presumably no drug-associated side effects. A few questions and answers give her a slight association with early dementia, but her suspicion is rapidly dismissed. She checks Peter’s toe, he takes off his right sock, and Kirsti observes a typical corn, easily remedied by self-care or a podiatrist. She returns to the headache, checking Peter’s blood pressure, which is normal. He seems relieved and does not want further investigation. Kirsti reassures him to return if the headache remains or gets worse.

Kirsti revisits urgency considering the probability of constipation as an early sign of cancer in a man aged 63. She asks Peter about his general condition, his eating habits, how often he goes to the toilet and if he ever noticed blood in the stool. Belly palpation reveals nothing special, and Kirsti initiates a small-scale investigation addressing the malignancy probability with blood and stool tests. She closes today’s attention to this part of the case and trusts that she will be reminded of the constipation issue when the test results appear.

During the dialogue about Peter’s general condition, he casually mentions that a friend recently died. Since then, he has not made his daily walks. This comment draws Kirsti’s attention to his mood - is he depressed? After probing with a few words, she understands she is on the wrong track. Peter’s talkativeness and distress, however, indicate to Kirsti that something indispensable might be embedded in mentioning the walks. Kirsti asks more specifically, and Peter tells about periods of weakness, sometimes accompanied by a jumpy heart. Kirsti feels some relief when this last piece of information materialises, and the indication of cardiac arrhythmia comes distinctly to her. Periodic atrial fibrillation is now confirmed by palpation of Peter’s pulse, auscultation of his heart sounds and, conclusively—the same day, at the practice laboratory—by a typical ECG pattern.

Peter leaves Kirsti’s consultation room 13 minutes after arrival. Several diagnostic ideas have been floating around in the consultation room and in the GP’s mind. Peter’s medical record for that day contains four diagnostic labels: a corn (which he himself will handle), constipation (low-key investigation initiated), headache (normal blood pressure) and atrial fibrillation (appointment next week to plan management). No mental illness has been pursued or confirmed. The dialogue was tranquil and reciprocal. Peter’s previous medication has been briefly reviewed and is not changed. Although some of the diagnostic paths have been straightforward, the medically most significant one, cardiac arrhythmia, was not.

## Analysis of diagnostic knowing, applying Lonergan’s theory and concepts to the case

Now, we shall apply Lonergan’s theory and concepts and demonstrate how these offer access to analysis of diagnostic knowing exemplified by the consultation presented above. Diagnosis starts with (1) the prereflexive experiences and imaginations of the GP related to the patient’s presentations. The GP transforms experiences and imagination into (2) questions for understanding. Eventually, (3) reflection judges the trustworthiness of diagnostic hunches or ideas, or they are just recognised without further probing.

First, Kirsti’s imaginations are activated with the *experiences* evoked by multiple sources of information. Over the years she has incorporated Peter’s image as a person, sensing that today he appears a little more distressed than usual. His appearance makes her more attentive to his mood. Peter presents verbal information about his bowel movements, headache, not going for walks anymore and several other issues. Kirsti checks his toe and notices the skin changes, adding these perceptions to her experiences applied for diagnostic knowing.

Then the experiences create images and interpretations, contributing to questions for *understanding*, such as whether Peter’s chronic conditions or medications are associated with his symptoms. Questioning may emerge as a transitory concern, not extensively ruled out, for example concerning Peter’s headache and mental state. Another example of how interpretations create questions is whether the constipation indicates cancer. Such questions for understanding activate a more determined awareness and interpretation of the complaints, involving for example the duration of the problems and which of the problems that deserve elaboration. Questions for understanding may trigger new questions adding to or replacing the understanding, such as the information that Peter gave up his walks because of weakness and palpitations.

Finally, when ideas and questions regarding diagnoses have been developed, they may be (but are not always) *judged* and subjected to reflection. The idea that Peter’s appearance might indicate early dementia is swiftly reflected upon and abandoned, without any formal judgment. But the surprisingly emerging hunch of cardiac disease leads to specific questions probing for a specific diagnosis or class of diagnoses, with the recognition of irregular pulse confirmed and specified by a pathognomonic ECG. Judging and reflecting upon the questions leads to the conclusive judgment that Peter suffers from episodes of atrial fibrillation. But a diagnosis of cardiac disease was considered initially neither by the patient nor the GP. Expanding upon diagnosis of the bowel problems, on the other hand, remains provisional by the end of the consultation, as some questions for understanding are still unanswered. Since these questions are attributed potential danger, the GP will follow up in due time. The diagnosis of the corn deserves no further exploration by questioning or judgment, and the headache is judged as insignificant based on insights drawn from questioning and examination of the blood pressure. The pattern of this consultation, illustrated by concurrent exploration of several different and potentially interacting complaints, demonstrates typical challenges in general practice. Diagnostic knowing is a dynamic and sometimes circular process, in which the different threads are not necessarily brought to a completed cycle of cognition.

*Inverse insight* occurs when Kirsti asks Peter about his general condition. His response is specific, including the information that he no longer makes his walks. Kirsti suddenly realises that the question she had probably asked herself – is this maybe because Peter mourns his friend and is depressed? – was faulty and contributed to a wrong track. This insight sharpened her attention for other imaginations, from which she understands that walks make Peter tired and his heart jumpy. After brief probing, a possible mood disorder was dismissed, and new questions may evolve. Kirsti considers a cardiac disorder which – although potentially serious – is quite easily investigated and diagnosed. Until the brief passage about walks, the flow of information in the consultation offered no indication of heart disease.

## Discussion

Lonergan staged his theory as a universal understanding of insight, with interpretative action as the integral element of knowing. Above, we have presented a theoretical analysis of a typical case of diagnostic knowing, demonstrating how Lonergan’s theory may support a systematic and transparent recognition of interpretative actions conducted by the GP, informed from multiple sources. Below, we discuss the strengths and limitations of such an approach to analysis of diagnostic knowing.

### What is known from before, and what does our analysis add?

We are not the first to address diagnostic knowing, neither in general [10,22,23] nor in the general practice context [5,13]. Interpretation has also previously been highlighted as a clinical skill [16], emphasizing for example the general practice interaction and perception of cues [24]. Applying Lonergan’s theory and concepts for analysis of diagnostic knowing in general practice, our presentation complements the pursuits of Engebretsen et al. [19], contributing to make medical knowing accessible for analysis and reflection.

Formulation of questions and choice of adequate strategies for the pursuit of the relevant evidence for the particular case are significant dimensions of clinical knowing [14], aligning with the original understanding of evidence based medicine (EBM) [25]. The particularity of diagnostic knowing that we reveal, is however, incompatible with the basic idea of generalizability of sources for knowing and standardisation of evidence in contemporary EBM [26]. Understanding diagnostic work as *hypothesis testing* implies that a limited number of hypotheses are established early in the diagnostic process, guiding data collection, whilst *pattern recognition* infers immediate categorisation of new cases comparingwith previous instances or abstract prototypes [11]. Our case includes indeed both these modes – pattern recognition demonstrated by the GP’s categorisation of Peter’s toe problem as a corn, and hypothesis testing considering cardiac disease with confirmation of atrial fibrillation. The latter path, leading to the atrial fibrillation diagnosis through hypothesis testing, also demonstrates experiencing, understanding, and judging, Understanding how the doctor arrived at this hunch can, however, only be recognised through a lense of inverse insight.

Our mission is not to translate processes such as hypothetico-deductive reasoning into a new language. We want to demonstrate how Lonergan’s perspectives offer access to complex cognitive processes of diagnostic knowing, where the inevitable subjectivity of interpretation is acknowledged without dismissing scholarly rigour. Interpretation is an integrated, positive aspect of diagnosing that can be fostered and developed [18], not only a subjective bias in cognition [11]. Interpretive action is needed to create hypotheses for deduction as well as questions for understanding.

The case highlights how a medically significant diagnosis (atrial fibrillation) may evolve otherwise than a deductive consequence of complaints primarily presented by the patient. Analysis shows how diagnostic knowing initiated by interpretation of multiple subtle presentations is elaborated towards different aspects and levels of knowing. The opening lines from the patient function as ideas of where to look for possible explanations of the patient’s complaints rather than diagnostic hypotheses. In Lonergan’s terms, such ideas serve as ‘questions for understanding’, for example ‘Could this complaint represent side effects of the patient’s medications?’, or ‘Are the presented problems associated with the patient’s recent retirement?’ The patient’s distress draws the GP’s attention to parts of the story associated neither with the patient’s reasons for the encounter nor his presented complaints. Distress does not, however, work as a hypothesis but rather as an unspecific sensation of something wrong. Questioning the distress is done before the doctor pursues and arrives at a specific diagnosis. The patient’s complaint, constipation, is not ignored but pursued towards adequate levels of understanding. Our analysis exposes how different processes of diagnostic knowing are entangled and can still be reflected upon.

*Heuristics* are efficient cognitive processes based on practical rules of thumb and different from analytics [15], but this concept does not adequately cover the cognitive phenomena of diagnostic knowing we have described. Daly argues that *understanding* is distinct from *intuition* in processes of learning from experience [4]. *Gut feelings* have also been proposed as the third track of GPs’ clinical reasoning, being a particular type of intuitive feelings, usually confined to prognostic assessments of the patient’s situation and often accompanied by bodily sensations in the doctor [27]. Neither does this concept match the different aspects of diagnostic knowing that we have portrayed above, especially not the obligation of interpretation combined with reflection. On the other hand, Lonergan’s concepts offer specific access to unpack the contents of the ‘black box’ of gut feelings.

Lonergan’s theory reveals diagnostic knowing with adequate insight achieved also at levels of understanding less advanced than formal judgment. The relevance of the questions determines the adequate level and value of a diagnostic conclusion. Lonergan’s theory embraces insight about multiple problems presenting at the same time, a typical diagnostic challenge for the GP. Furthermore, our analysis demonstrated how situations involving several complaints, diseases and medications encompass appropriate trade-offs between problems deserving urgent and extensive attention versus problems that more easily reach a resolution or can wait.

Resistance towards shifting from one hypothesis to another is one of the persistent problems of clinical reasoning [11]. In our case, the GP rapidly dismisses the idea that Peter is depressed and, during the talks about walks, suddenly gets the inverse insight that she pursued the wrong question. Recognising this made it possible for her to shift attention and approach an awareness for a possible heart disease. Lonergan argues that inverse insight does not occur often [18, pp 43–50]. Trusting our experiences from clinical practice, however, we suggest this phenomenon to be an important and frequently occurring aspect of diagnostic knowing, deserving more attention. A reflective approach, including a capacity for judgment of one’s own insights, can make it easier for the GP to abandon a stray idea and admit that s/he is ‘barking up the wrong tree’ [14,19].

### The context of general practice

Engebretsen et al. were the first to apply Lonergan’s perspectives to medicine, discussing decision making in the contexts of respectively evidence-based medicine, trauma team and physiotherapy [17,19,28]. Later, Daly presented an oncological case [4]. These contributions encouraged us with a strong impact on our approach, while we also noticed that these situations involve diagnostic challenges very different from those in general practice. Supported by Lonergan’s theory, we therefore set out to explore the preconception that diagnosis in general practice is a special instance of the structure of all human knowing. Still, we do not expect Lonergan to cover all relevant features for a specific setting. Although our analysis demonstrated a capacity to include a broad range of issues with a special impact for the GP, such as time, organisation, doctor–patient relationship and the complex pattern of morbidity, we noticed that certain unique aspects of diagnostic knowing are not fully covered by Lonergan.

*Time* is a diagnostic asset for GPs encountering patients’ complaints and diseases in all stages of their natural history [5,29]. Some health problems are self-limiting and disappear within a short time, whereas others will evolve from vague sensations towards distinct patterns of symptoms and disease, and still others will remain medically unexplained, although the burden of suffering is substantial [9,30]. In general practice, diagnostic knowing often comprises a specific approach: to wait and see if a symptom develops as significant [13,31]. It also entails the possibility that the GP revisits and interprets initial experiences of the patient’s problems differently. In our case, analysis demonstrates how time is utilised when the GP plans the follow-up. She tolerates a provisional level of insight and uncertainty with Peters headache, as she will soon see the patient again, and a completed cycle of cognition is neither relevant nor necessary.

The *organisation* of general practice also has an impact on diagnostic knowing. The GP finishes the consultation within a limited number of minutes, and the possibilities of referral for further investigation vary depending on geography and the services required. In our case, the GP’s laboratory offers an important temporary diagnostic insight determining the need for further investigation. Anaemia or faecal blood would indicate urgent colonoscopy, whereas the absence of such pathology offers a time span to observe the course of illness. In this way, the organisation frames diagnostic knowing to settle with locally available tests, using progress over time to estimate the need for further evaluation.

The *doctor–patient relationship* is a specific feature of general practice, often developed over years, transcending individual episodes of illness [5]. Continuity of care furnishes diagnostic knowing with presentations from experiences inviting to interpretation and judgment. The GP can see the patient again to solve more problems, thus establishing diagnostic knowing consecutively by integrating new insights. Furthermore, observation over time may clear the way for knowing when the situation is not urgent. In our case, the GPs prior knowledge of the patient included an overview of his medical history, enhanced the exchange of knowledge, and sensitised the GP’s awareness for changes in the patient’s demeanour and capacity. We realise that such preconceptions, representing vital sources of information for diagnostic knowing, are not easily grasped with Lonergan’s concepts. *Values* (those of the GP, the patient, the family, and the culture) are also involved in the diagnostic approaches and in subsequent goals and actions when the long-term relationship may transcend facts and logic.

The morbidity pattern in general practice contains many *unsorted problems and complaints*. The presented problem often lacks a clear-cut answer [31], and diagnostic knowing does not always lead to a diagnosis with the capacity to explain or cure illness. As demonstrated in our analysis, multimorbidity represents particular challenges, with symptoms from different diseases mixing, merging and staging in aberrant formats, often at the same time [32]. The GP must consider which information is noise or conversation, and what really matters for the patient’s health. Reflecting upon questions for understanding whilst assessing and prioritising which of them deserve further attention for understanding and judgment, supports the process of sorting out. Simultaneous management of multiple problems requires skilful diagnostic knowing. We have shown how Lonergan’s theory and concepts make it possible for us to decode and recognise different elements of the cognitive sequences where prior judgments call forth new insights, verifications, value judgments and decisions. In this way, we have tried to contribute to make the process more accessible for reflection and learning.

### Theory for enhanced understanding of practice

The clinical encounter is constitutive of general practice, and the specific knowing generated in this encounter deserves status as *evidence* [14]. Daly argues that ‘methodical attention to the structured sequence of cognitive and deliberate operations is itself a potential resource both in clinical practice and in coordinating the development and implementation of diagnostic, prognostic, and therapeutic tools’ [4]. Regarding diagnostic knowing as interpretation embedded in subjectivity, we commit ourselves to *reflexivity* towards this specific mode of knowing, rather than trusting an algorithm. Presenting our analysis of the case, we have demonstrated how Lonergan’s theory and concepts helped us organize our analysis of diagnostic knowing and discuss the impact of this. For GPs, knowing your own knowing is essential for the appropriate and trustworthy act of reasoning. Reflexivity is therefore a crucial diagnostic skill for GPs, offering an opportunity for alternative interpretations, new questions for understanding and, sometimes, inverse insight [7,14]. In this way, systematic analysis may reveal the content and meaning of clinical knowing and acknowledge the potential for sharing and critical discussion [14].

### Strengths and limitations of our approach

The case that we established for analysis embodies exemplary elements of diagnostic knowing in general practice. The core elements of this case with the patient, his complaints, and diseases, as well as the course of the consultation are recognisable for a broad range of GPs across nations, cultures, and health care systems. The GP we portrayed was an experienced practitioner, and the doctor–patient relationship has been longstanding. We do not know whether a less experienced GP would have perceived all the diagnostic clues presented here and interpreted them similarly. Nevertheless, these qualities support the claim that our analysis and findings bear a strong potential for transferability. On the other hand, our presentation is also intended as an interpretative contribution. A qualitative study with participant observation would give access to elements beyond those presented here.

### Implications for clinical and academic practice

Our analysis sustains the idea that conceptual tools can enhance doctors’ awareness, thinking explicitly about what they are doing when diagnosing. We found Lonergan’s theory especially useful to understand diagnostic knowing in the complexity of general practice. Still, several aspects of the theory as well as our analysis, may be transferable to other medical contexts. Our analysis demonstrates promising capacity for transparent and reflexive approaches to diagnostic knowing, with a strong potential for clinical teaching and further theoretical reflections. Our analysis encourages us to argue that an interpretative understanding of diagnosis will complement hypothetico-deductive strategies by recognition of substantial diagnostic modes, thereby changing clinical practice by giving access to scholarly reflection and teaching.

## Ethical approval

For reasons of confidentiality, we constructed the case by assembling medical and demographic details from several instances, arguing that a consultation with similar elements of diagnostic knowing is familiar and frequently occurring in general practice. All procedures performed in studies involving human participants were in accordance with the ethical standards of the institutional and/or national research committee and with the 1964 Helsinki declaration and its later amendments or comparable ethical standards. Since the article does not contain studies with actual human participants, informed consent and approval from The Regional Committees for Research Ethics was not relevant.
